# Effect of a home-based physical rehabilitation program via virtual reality on the functional outcomes of frail older adults: a quasi-experimental study

**DOI:** 10.1038/s41598-025-88225-8

**Published:** 2025-02-04

**Authors:** Doaa Mohamed Zein El-AbdeenMohamed, Heba Noshy Abd EL-Aziz Mohamed, Soad Hassan Abd Elhameed

**Affiliations:** 1 Assistant Lecturer in Gerontological Nursing Department, Faculty of Nursing, Mansoura University, Mansoura, Egypt; 2 Assistant Professor in Gerontological Nursing Department, Faculty of Nursing, Mansoura University, Mansoura, Egypt; 3 Professor in Gerontological Nursing Department, Faculty of Nursing, Mansoura University, Mansoura, Egypt; 4 Acting Dean of the Faculty of Nursing, of New Mansoura University, Department of Community Health Nursing, Faculty of Nursing, New Mansoura University, New Mansoura, Egypt

**Keywords:** Effect, Home-based, Physical rehabilitation, Virtual reality, Functional outcomes, Frail older adults, Diseases, Health care, Medical research, Ageing

## Abstract

Given the rapid aging of the population in Egypt, efforts to slow down or prevent frailty. Virtual reality technology constitutes a promising rehabilitation strategy, but its effect on frailty in older adults remains inconclusive. A non-equivalent control pre, post, and follow-up test design was used with a sample of 70 prefrail or frail older adults. In 3 urbans affiliated to Dakahlia governorate, tools of data collection; Mini-Cog, Structured Interview Schedule, Survey of Health, Aging and Retirement in Europe of the Frailty Indicator (FI), Short Physical Performance Battery, and Falls Efficacy Scale. For each group, a significant improvement in frailty, physical performance, and falls from each measurement period to immediately after (*p* < 0.001), and within the 30-days study period while those in the control group were relatively stable over time and sometime worsen. The baseline characteristics and assessment results were similar between groups. We observed significant improvements in the intervention group in terms of frailty criteria, standing balance, gait speed, chair stand, and fear of fall. No improvements were observed in the control group, we anticipate a decrease in frailty index, and increase in standing balance, reinforcing the proven benefits of the exercise in this vulnerable population.

## Background

People 65 years of age and over make up 9.3% of the world’s population. This fast aging of the population is especially noteworthy since aging causes a significant reduction in both physical and mental capacities, which results in frailty. People who are frailer tend to be more prone to certain ailments, which has a negative effect on total healthcare expenses. The aging-related deterioration in physiological processes, the body’s diminished reactivity to external stimuli, and the heightened risk of illness and disability are the hallmarks of frailty. Frailty progression can also lead to the development of diabetes, heart disease, and muscle loss by interfering with vital systems such the respiratory, cardiovascular, endocrine, and musculoskeletal systems^1^.

The term “frailty” refers to a clinically recognized condition of greater vulnerability in older individuals that is brought on by age-related decreases in physiologic reserve and function across several organ systems, making it more difficult for them to cope with daily stressors or acute ones. The frailty phenotype (FP), also known as Fried’s definition or the Cardiovascular Health Study (CHS) definition, and the frailty index (FI) have also become prominent definitions with suggested evaluation techniques based on this conceptual framework throughout the previous ten years^2^.

To give frail older persons the best possible clinical treatment, doctors need to be aware of the many symptoms, indicators, and negative outcomes that are linked to frailty syndrome. Clinically evident in the end stage, frailty is frequently recognized by signs of repeated falls and injuries, impairment, vulnerability to severe sickness, and a poor recovery from acute stress^3^.

The only interventions that have been shown to enhance clinical outcomes in frail older persons are exercise and geriatric multidisciplinary evaluation and treatment models. With increased knowledge of the scientific underpinnings of frailty, new approaches to geriatric care as well as more efficient treatment plans focusing on certain physiologic systems are anticipated^4^.

Exercise-based therapies have the potential to stop, slow down, or even reverse the onset of frailty. Therefore, it has been suggested that more research on intervention in fragile populations is necessary. Elder folks are clearly trainable. A recent systematic study on the advantages of progressive strength training found that training increases muscle strength^5^.

Regular exercise has been shown in prior research to slow down the aging-related decline in physical function, enhance muscle strength and balance, and improve other functional challenges. These benefits have been measured with measures like a lower timed up-and-go test (TUG), a higher Berg Balance Scale (BBS) score, and an improved Fall Efficacy Scale (FES-I) score^6,7^.

The World Health Organization defines rehabilitation as the use of strategies intended to lessen the consequences of impairment and incapacity while enhancing functioning to maximize social reintegration. The International Classification of Functioning, Disability and Health is closely related to the idea underlying contemporary rehabilitation (ICF). The ICF states that an illness or sickness may dysregulate not just a few organs but also functions and activities, which might impact an individual’s involvement in the activity framework in addition to environmental and personal variables^8^.

Although virtual reality (VR) technology is a potential approach to rehabilitation, there is currently insufficient data to determine how it affects frailty in older persons. Thanks to recent developments in information and communication technology (IT), augmented reality (AR) technology is now being used in the field of fitness^9^.

To the best of what we know, despite these drawbacks, there is still a dearth of comprehensive reviews of observational or interventional research designs in the literature that describe the use of technologies in studies aimed at assessing and treating aging-related syndromes in the older adults^10^.

With the use of virtual reality technology, users may simulate exercise and experiences in a realistic setting. This technology is inexpensive and engaging, and it enables users to engage with many senses and receive real-time feedback. Through wireless interface sensors, a number of commercial virtual gaming systems, including the Microsoft Xbox (Kinect), Nintendo Wii sports system, and Dance Revolution System, can identify changes in player activity and movement in the real environment^11^.

Consequently, in the 3D setting, players may receive rewards, feedback on their actions, and instantaneous feedback to increase their motivation and will to continue exercising. However, because previous study used inconsistent and diverse selection criteria, it is still unclear how well VR gaming therapies affect physical function, balance, and falls in the senior population. According to research, the control group, which got conventional treatment alone, outperformed the VR intervention group, which trained with virtual games, on the TUG exam^12^.

Physical exercise and sarcopenia prevention should be the top priority to promote healthy aging and manage an aging population. Loss of muscle mass, shape, and strength is linked to aging and inactivity. The annual rate of muscle loss for people over 50 is 1–2%^9^. Exercise and gaming are combined in home-based exergame programs, which are becoming more and more popular as a means of motivating people to engage in physical activity at home^9^.

The suggested method of rehabilitation is to use the ICF scale classifications to determine the person’s initial level of frailty. Furthermore, it is commonly recognized that older adults may not be aware of the functional, psychological, and social implications of their own health. For this reason, conducting a comprehensive geriatric assessment (CGA) may be essential. This process identifies medical, psychosocial, functional, and environmental issues and develops a comprehensive therapy and assessment plan with the goal of enhancing patients’ health as a whole^13^.

## Methods

### Study design

A Quasi experimental research design with a nonequivalent control group pretest-posttest type was utilized in this study^14^.

### Study setting

This study targeted 3 urban areas affiliated with Dakahlia Governorate to produce nationally representative samples. According to ‘’Population Clock’ announced by the ‘Central Agency for Public Mobilization and Statistics’’, the Dakahlia governorate was one of the largest Egyptian governorates in terms of population, as it ranks fourth in terms of population, with a population of approximately 7 million, 32 thousand and 560 people; at a rate of 6.8% of the total population of Egypt, the percentage of older adults aged more than 65 years was 4.31%, with 4.68% in urban areas (Central Agency for Public Mobilization and Statistics, 2023). Three of Dakahlia’s eighteen centers are urban areas: Mansoura, the capital city with a population of around 439,000; Dekernes, the city with a population of about 167 thousand; and Mitt-Salsil, a city of about 31,000 people.

### Subjects

#### Sample technique

A cluster sampling technique was used, and 70 community-dwelling older adults living in the abovementioned setting were included in this study. First, the three urban areas in Dakahlia governorate were divided into 15 clusters (5 from each city), and each section or cluster was randomly chosen by the software system ‘randomizer’ https://play.google.com/store/apps/details?id=com.giannis.randomizer&hl=en. Second, the oldest regions were chosen by first selecting a direction at random from the city’s major landmark, and then selecting a direction to begin with and follow (e.g., by spinning a pen). Third, a residence was selected at random to serve as the initial sample house after the number of structures in that direction was tallied. Until all clusters were covered, the next house was selected by going to the closest adjoining house.

The above-described random selection of the path from the central starting point was followed to contact homes until all necessary data about each cluster was obtained. The study group (A) and the control group (B), which were matched, were formed from the participants and were administered through households to the target population in the previously indicated settings. These groups met the following requirements:

- Based on Fried’s criterion, older adults over 60 years of age were classified as frail or prefrail.
- Higher Mini-Cog scores were indicative of normal cognitive function.
- The participants had good visual acuity and hearing acuity and were willing to participate in the study voluntarily.
- Lack of prior Nintendo Wii Fit Plus^TM^ (NWFP) experience.
- Able to move independently and carry out physical activities in an orthostatic stance.

#### Sample size calculation

The sample size for studying the functional outcomes of Nintendo Wii Fit Plus TM for frail older adults was calculated via research software (https://clincalc.com). Similarly, a previous study^15^ revealed a pronounced improvement in gait in the intervention group (before, after training, and follow-up assessment), with mean SDs of 15.7 ± 4.59, 18.8 ± 5.75, and 19.5 ± 6.12, respectively, compared with 17.1 ± 6.32, 16.9 ± 5.82, and 18.6 ± 5.93, respectively, in the control group. At Power (1−β error probability) = 0.80 and α error probability = 0.05. The sample size required was 64 older adults, and 10% was added because of drop out. Therefore, the final sample size was 70 older adults (35 older adults in the VR intervention group and 35 older adults in the nonintervention group).

#### Tools of data collection

Five instruments were employed to gather relevant data for the research:

##### Tool I: Mini-cog

It is a composite of three-item recalls and clock drawings, was developed by^16^ as a quick test to distinguish between those with dementia and those without in a population sample of diverse older individuals. It is an oral cognitive screen with five points that includes three-word recall (0–3 points), a clock-drawing activity (0–2 points), and three-word registration (0–0 points). A higher score indicates greater cognitive function.

##### Tool II: demographic and health-related data structured interview schedule

It was developed by the researcher after a review of the relevant literature^17,9^ and is divided into two parts:

Part 1: The socioeconomic characteristics of senior citizens include age, gender, marital status, educational attainment, employment prior to retirement, income, and housing circumstances.

Part 2: Health-related data, including medical history of chronic diseases, medication intake, previous hospitalization, history of falls in the previous year, and unhealthy behavioral lifestyles, such as smoking, were collected.

##### Tool III: Survey of Health, Aging and Retirement in Europe of the Frailty Indicator (SHARE FI)

This tool was originally developed for primary care settings by^18^ and validated by^19^ to assess frailty using one objective handgrip strength (kg) assessment utilizing a dynamometer and four self-report questions (fatigue, weight loss, slowness, and poor physical activity). The left and right hands were measured twice in a row, and the highest extent out of the four was chosen. Frailty was classified into three groups based on the total number of different characteristics that were fulfilled^20^.

- Score 0: not frail.
- Score of 1 or 2: prefrail,
- Score 3, 4 or 5: frail.

##### Tool IV: short physical performance battery (SPPB)

This tool was developed by^21^. The three tests are to walk (between 2.4 and 4 m), balance (three positions: feet together, semi tandem, and tandem), and get up and sit in a chair five times. The tests have to be completed in the correct order since, if the patient begins by sitting and standing up, they may become weary and provide misleading negative results on both of the additional subsection^22^.

Three elements made up the scoring system: the time it took to finish a 3- or 4-meter walk; the ability to stand for up to 10 s with one’s feet positioned in three different ways (side-by-side, semi-tandem, and tandem); and the amount of time it took to get up from a chair five times. The ability to maintain balance in each of these positions determined the score for the standing balance tests. In the other two assessments, points were awarded based on two factors: first, the time it took to do each activity, and second, the ability to execute the tasks. Each assignment carried four points, and the combined results of the three assessments produced a final score that ranged from 0 to 12, with 4 serving as the lowest and 12 as the highest. A higher score denotes a greater function level, whereas a lower score denotes a lower function level.

##### Tool V: falls efficacy scale - FES

This scale was developed by^23^ and translated into the Arabic language by^24^. The scale measures the degree of anxiety associated with the potential for falling during ten activities of daily living, including dressing and undressing, cooking, bathing or showering, climbing and descending stairs, cleaning the room, sitting or standing up in a chair, opening doors or answering the phone, shopping, bending or lifting objects to get something, and going outside. Test-retest reliability was good for the FES (*r* = 0.70).

From 1 (not at all bothered) to 4 (severely concerned), each question was given a rating. The overall score is split into the following categories, ranging from 10 to 40:

- Not at all ranged from 1 to 10.
- Slightly concerned from 11 to 20.
- Moderately concerning from 21 to 30.
- Very concerned from 31 to 40.

### Data collection process

#### Phase I: preparatory phase

Permission to do the study, a review of the literature, the creation of study instruments, and tests of the tools’ dependability and content validity were all included in this step. This phase ran from the beginning of November 2023 until the end of December 2023, a total of two months. The following steps made up this phase:

### Administrative stage

After the objectives and methods of the study were outlined, permissions to perform the investigation were acquired from the relevant authorities of the study setting.

study of the literature: To understand the several facets of the research subject, this involved a study of the previous and current relevant literature and studies, utilizing books, periodicals, magazines, and articles that are readily available. creation of the Nintendo Wii Fit Plus^TM^ (NWFP)’s navigation and study aids. Nintendo Wii Fit Plus^TM^ and the study instruments have chick validity (NWFP).

The content validity of the study tools (I, II, III and V) was evaluated and amended by a group of five gerontological nursing specialists. To determine how closely the scores reflected the factors they were meant to assess, the tool’s clarity, substance, question order, and relevance/irrelevance of material were all updated. The jury members’ recommendations were implemented. Tool IV: The researcher translated the SPPB into Arabic, and an English language specialist from the Faculty of Education verified the accuracy of the translation. In this investigation, a backup translation approach was employed to guarantee the accuracy of the translation.

#### Reliability of the study tools

In this study, the Arabic versions of the Mini-Cog tool III, tool V, the Falls Efficacy Scale (FES), and the Survey of Health, Aging, and Retirement in Europe of the Frailty Indicator (SHARE FI) were utilized. The Cronbach’s alpha test was employed to assess the data (α = 0.834, 0.963, and 0.851, respectively).

The internal consistency of tool IV, the SPPB, was tested via Cronbach’s alpha coefficient test and was found to have a consistency of α = 0.934.

#### Phase II: the exploratory phase

The survey included a pilot study and fieldwork:

##### Pilot study

To show the viability and application of the tools and to gauge the time required for data collection, pilot research including 10% (7) of the total individuals was conducted. The research did not include the older persons who participated in the pilot project.

##### Work domain

This phase lasted four months, commencing in January 2024 and ending in April of the same year. The following steps made up this phase:

Three days a week, for eight hours a day, the researcher was in the previously chosen location. After providing written consent to participate in the study, after the researcher explained its purpose and nature, study subjects who met the sample criteria and consented to be involved were interviewed one-on-one. The interview began with the researcher introducing herself and explaining the purpose of the study to collect the necessary data via all study tools.

**Fig. 1:**
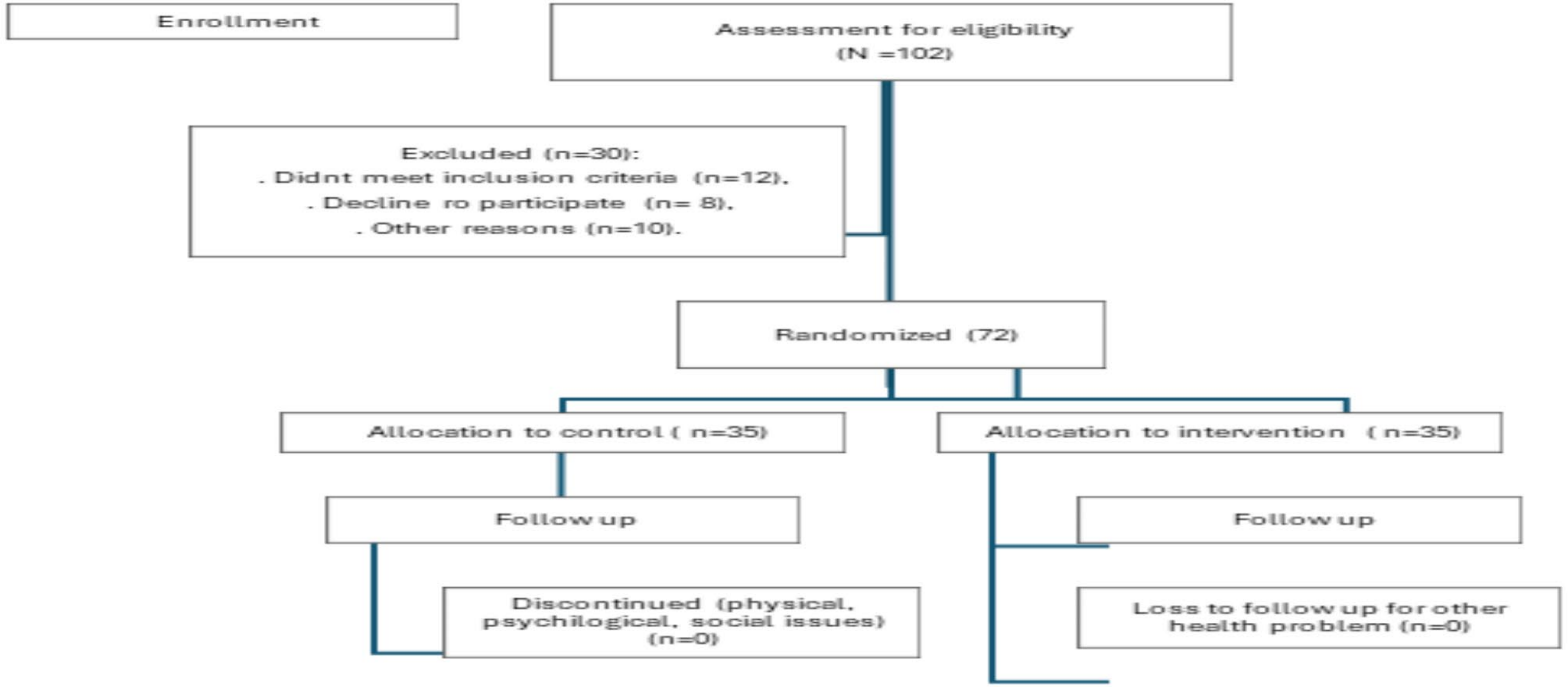
Assessment for eligibility and enrollment.

The methodology of the research was implemented in the following four phases:

1. Measurement phase: All the study tools were used to ensure that all the older adults were screened to confirm that they fulfilled the inclusion criteria.

2. Planning phase: Using the block randomization procedure (split into block A and block B), older persons were randomized to either the control group (control; *n* = 35) or the intervention group (Wii group; *n* = 35). In this sense, the Wii group and the control group both had the same number of older individuals (See Fig. 1).

- The control group received no intervention other than the usual distribution of colorful pamphlets promoting the advantages of exercise for fragile older individuals, whereas the older adults in the Wii group received virtual reality-based rehabilitation activities. Each game lasted between two and three minutes. It took around three minutes to go from one game to another. If a participant could not finish the session, a make-up session might be scheduled for the same week.
- A USB strap that was attached to a smartphone or smart TV allowed users to play the Nintendo gaming console on YouTube and VR glasses. We chose games that tested lower-extremity muscular strength and balance from the Wii Fit software. This is applied by the system to the virtual world that is seen on the screen. In addition, it was shown on the screen, and the player followed the movement, offering sensory input via vibrations in response to the user’s varied motions in addition to aural and visual feedback. Experts in the dynamics of exercise for older adults came to a final choice based on their combined opinions.
- The exergame routine of a 10-minute warm-up, a 10-to 15-minute workout, and a 10-minute cool-down.

3. Implementation phase: Prior to using the VR application to gather baseline data using the research instruments, each older adult was interviewed separately.

- An older adult is asked to wear VR glasses, as it displays images of movies and games at a three-dimensional stereoscopic, 360-degree viewing angle, you may experience the event as though you are within it in all its aspects and feel anxiety, excitement, and intrigue based on what you see in the games or video.
- For real-world technology to work properly, a PC, laptop/smartphone/TV or console, a chair and VR glasses, in the beginning, of the console quickly process the data from the game or video clip and send that data to the virtual reality glasses to show the image smoothly when the internal components of the computer are weak.
- We discover that the pictures are split into two separate displays from the inside, each with a different display resolution based on the caliber of the glasses being worn. The images are shown at a minimum 120-degree viewing angle from the laptop or TV. Thus, when moving ahead to any side, the image displayed on each screen separately tracks the movement and displays the image in the desired direction and relies on determining the direction of the head on the “gyroscope” technology that rotates 360 degrees.
- The warm-up exercise portion consisted of stretching and a leg massage using a massage ball (Kieba Massage Lacrosse Balls) available at https://chiliguides.com/guides/guide-to-buying-a-massage-ball/(a massage ball is a piece of fitness equipment used for myofascial release). Myofascial release is a form of alternative therapy that helps relieve tension in muscles by breaking up scar tissue. It is suitable for older people who perform any physical activity or have stiffness in their muscles.
- The four phases of each session were completed by the participants in the same order: (I) The players were briefed on the rules of the two games that were going to be played during that session. (II) An aerobic-type game such as “Just Dance - Pump Up the Just or Hula Hoop” was played at https://www.bing.com/videos/riverview/relatedvideo?q=Wii%20Fit%20%20Torso%20Twist%20&mid=683B0DEB3901178069FC683B0DEB3901178069FC&ajaxhist=0 & https://www.bing.com/videos/riverview/relatedvideo?q=Wii%20Fit%20%20Torso%20Twist%20&mid=55A4B2B6FC84155BFB3A55A2B2B6FC84155BFB3A&aegypti=0. To replicate the motion of the hula hoop, players in this game were instructed to form smooth, wide-radius circles with their hips. To further improve implicit balance, participants were advised to elevate their arms to shoulder height.
- The adventure mode was chosen for the workout section and digital therapeutic exercise elements. The purpose of the design was to provide the participants with a virtual reality experience as they carried out different activities. (III) Competitions were held. (IV) Everyone in the group had to select a game to test out or play for five minutes to wrap up the session. It was anticipated that the video games would regain their lighthearted element in this last round.
- The cooling-off phase included breathing and stretching techniques. The researcher made sure the residence was conducive to physical activity before starting the exercise program by measuring the distance from the smart TV and phone and locking up the workout space.
- Furthermore, the investigators put console devices in the homes of each senior participant and instructed the participants and their family caregivers on how to operate them. At last, the investigator placed a non-slip mat on the ground and walked participants through each activity. The participants were instructed to cease exercising and get in touch with the researcher if they experienced any physical discomfort, pain, dizziness, or exhaustion. The participant’s phone number or text message following the exercise was the last request made by the researcher.
- No exercise regimen was given to the control group participants for eight weeks, and the outcomes of the posttest were unaffected by the exercise regimen.

4. In the evaluation phase, older adults’ outcomes were evaluated as postintervention in both groups. All the older adults were assessed on three occasions via tools III, IV and V before and immediately after the implementation of the program and 30 days after the end of the program (follow-up)^15^.

The potential adverse events may include:

- *Physical Injuries*: Participants may be at risk of falls or strains due to unfamiliarity with virtual reality equipment or physical exercises. *Psychological Effects*: Some individuals might experience discomfort related to virtual reality experiences. *Technical Issues*: Malfunctions with the virtual reality equipment could result in frustration or confusion for participants.
- Therefore, Safety Management Considerations including conduct thoroughly assessments of participants’ physical and psychological health to identify any pre-existing conditions, provide comprehensive training sessions on the use of virtual reality to raise his/her familiarity, regular check-ins to identify any adverse events early on, establish clear conventions for addressing any adverse events that occur during the study. Clearly communicate potential risks during the informed consent process.

### Ethical considerations

The Mansoura University Faculty of Nursing’s Research Ethics Committee granted ethical permission. Every older adult included in the trial was fully told about the purpose, nature, risks, benefits, and remuneration before obtaining their written informed permission. Participants’ privacy and the confidentiality of the information gathered were guaranteed, and the data were utilized only for the objectives of the study. Every older adult who participated in the research was given the assurance that it was completely voluntary and that they may leave at any moment, without incurring any fees or punishments.

The current study had two limitations: first, it only included a small number of participants; second, because the participants were exclusively older persons residing in Egypt’s metropolitan areas, it was challenging to generalize the study’s findings. Future investigations are required to determine how older persons with fracturs respond to virtual reality exercise on the Nintendo Wii Fit.

### Statistical analysis

Version 22 of the Statistical Package for Social Science (SPSS) was used to analyze the data. The first test for data normalcy was a one-sample Kolmogorov-Smirnov test. Whereas categorical data were presented as numbers and percentages, continuous variables were presented as means and standard deviations. Furthermore, inferential statistics were employed; The Chi-square test was used for two group comparisons in term of baseline demographic and health-related data. Additionally, Student t-test was used in intergroup comparison in terms of outcomes, the repeated measures ANOVA test was employed to evaluate the magnitude of the difference between two groups, utilizing the measure of effect size known as partial eta squared (η2). In cases where the chance of mistake was less than 5% (*P* < 0.05), the results were deemed significant.

## Results

Table 1 shows the personal data of the frail older adults in both groups. Males were more prevalent (approximately 65.7% and 62.9%, respectively) in the intervention and control groups, and 54.3% and 57.1%, respectively, in both groups were rural residents. In terms of the age of the older adults in the control group, 48.6% of the individuals in the intervention group ranged from 60 to less than 65 years, whereas 40.0% of those in the intervention group ranged from 70 years and older. In terms of marital status, 62.9% and 65.7% of the older adults in both groups were married. Secondary education was the highest percentage of education in both groups, with values of approximately 45.7% and 42.9%, respectively.

With respect to work before retirement, in the intervention group, 31.4% of the farmers were farmers, whereas 31.4% were housewives in the control group. Approximately 62.9% and 68.6% of the older adults in both groups reported not yet having this trait, and approximately 62.9% and 65.7% of those in both groups reported that they were not enough when asked about their monthly income. With respect to living conditions, 71.4% and 80.0%, respectively, of the frail older adults in both groups were living with their families. There were no statistically significant differences among the groups in any of the personal data, such as sex, residence, age, marital status, level of education, work before retirement, currently working, monthly income, or living conditions, with p values < 0.05.

Table 2: Frailty indices of the older adults in both groups at the three time points (before, immediately after, and during follow-up). Before the program, the frailty index in the intervention group was 2.88 ± 1.05, whereas that in the control group was 2.80 ± 1.08, with no statistically significant differences detected between the groups (*p* = 0.632). The frailty index in the intervention group decreased to 1.40 ± 0.49 compared with 3.02 ± 0.85 in the control group, and statistically significant differences were detected between the groups (0.001**).

Finally, during the follow-up, the frailty index in the intervention group was 1.80 ± 1.13, whereas it was 3.08 ± 1.19 in the control group, with highly significant differences (*p* = 0.008**). There were statistically significant differences in the frailty indices on the three occasions in the intervention group (p = < 0.001**, whereas there were no statistically significant differences in the frailty indices on the three occasions in the control group (*p* = 0.088). The partial eta square among the intervention group (ηp2 = 0.491) indicated that it had a large effect size, as it was < 0.14.

Table 3: Physical performance of frail older adults in both groups on three occasions (before, immediately after, and during follow-up). There were no statistically significant differences between the intervention and control groups before the program (*p* = 0.383). Nonetheless, in every physical performance category, including the chair stand test, gait speed test, and standing balance test, there were statistically significant differences (*p* ≤ 0.001**) between the intervention and control groups both immediately after and after the program. The total physical performance of the intervention group before the program was 6.4 ± 1.5, which increased to 9.69 ± 1.45 immediately after the program, whereas that of the control group before the program was 6.83 ± 1.38, after which it decreased to 6.70 ± 1.31.

Table 4: Fear of fall among frail older adults in both groups on three occasions (before, immediately after, and during follow-up). The fear of falling in the intervention and control groups was 20.34 ± 7.7 and 20.45 ± 5.89, respectively, before the program, with no statistically significant difference between the groups (*p* = 0.318). However, immediately after the program, the fear of falling in the intervention group was 12.80 ± 3.49, whereas it increased in the control group to 22.00 ± 6.08. In addition, the follow-up fear of falling was 13.63 ± 3.52 in the intervention group and 22.28 ± 6.25 in the control group, with a statistically significant difference between the two groups (*p* < 0.001**). The partial eta square among the intervention group (ηp2 = 0.495) means that it has a large effect size, as it is < 0.14.

Table 5 shows the correlation matrix between the study variables among frail older adults in the intervention group on three occasions (before, immediately after, and during follow-up). There was a statistically significant weak negative correlation between frailty and physical performance (before, immediately after, and during follow-up) (-0.194, -0.147, and − 0.404*, respectively), as an increasing frailty score was associated with a decreasing physical performance score and vice versa. There was a statistically significant moderate positive correlation between frailty and fear of falling (before, immediately after, and during follow-up) (0.583, 0.576**, and 0.258, respectively), as increasing frailty scores were associated with increasing fear of falling scores and vice versa.

**Table 1:**
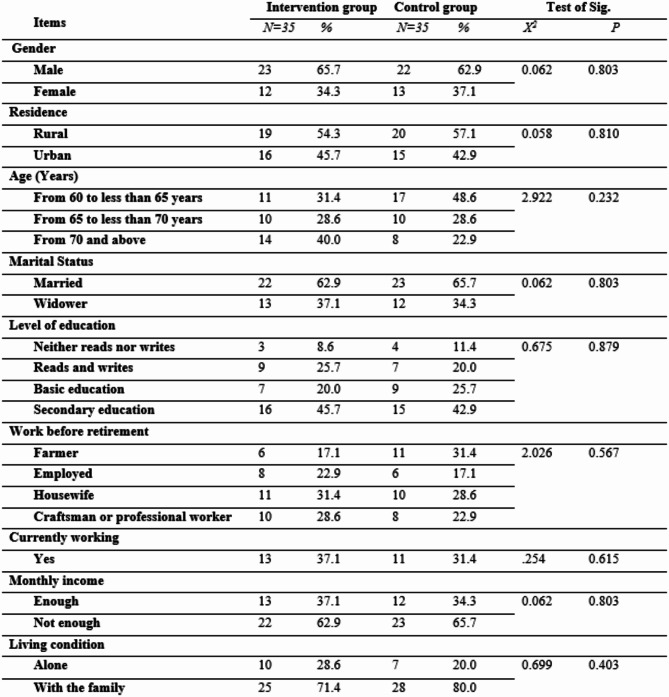
Personal data of frail older adults in both groups.

**Table 2:**
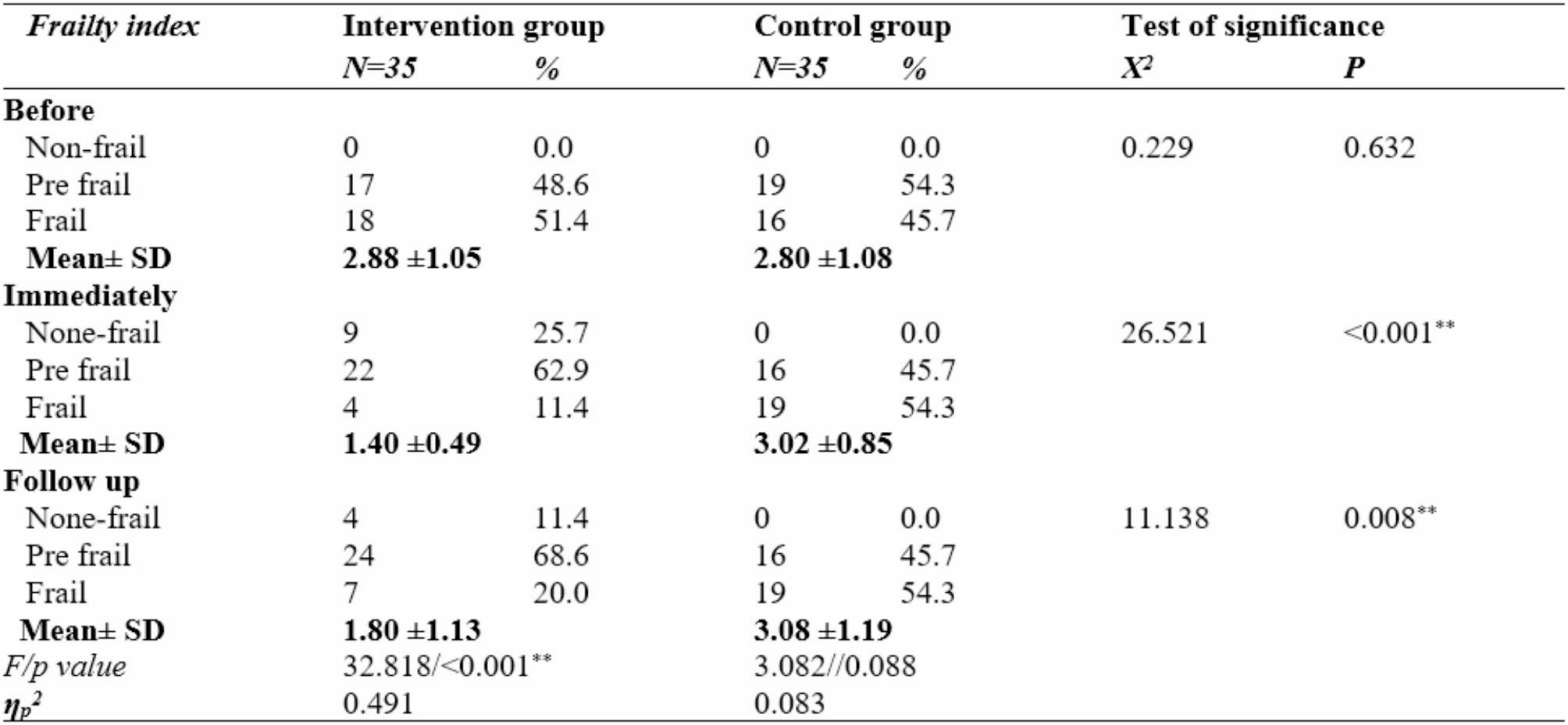
Frailty indices of frail older adults in both groups on three occasions (before, immediately after, and during follow-up). Partial Eta Squared (n²) = effect size. − 0.01 indicates a small effect − 0.06 indicates a moderate effect − 0.14 indicates a large effect.

**Table 3:**
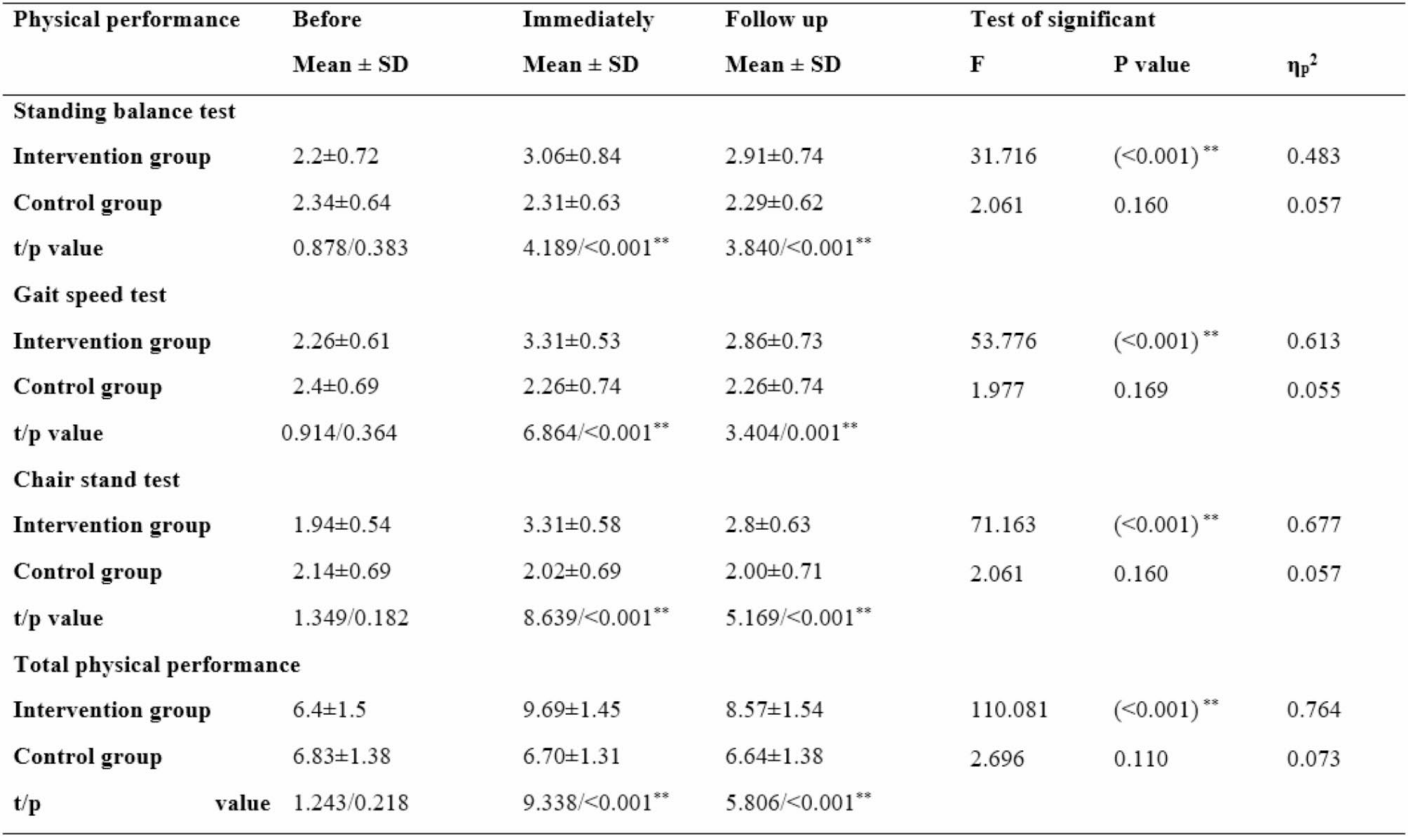
Physical performance of frail older adults in both groups on three occasions (before, immediately after, and at follow-up).

**Table 4:**
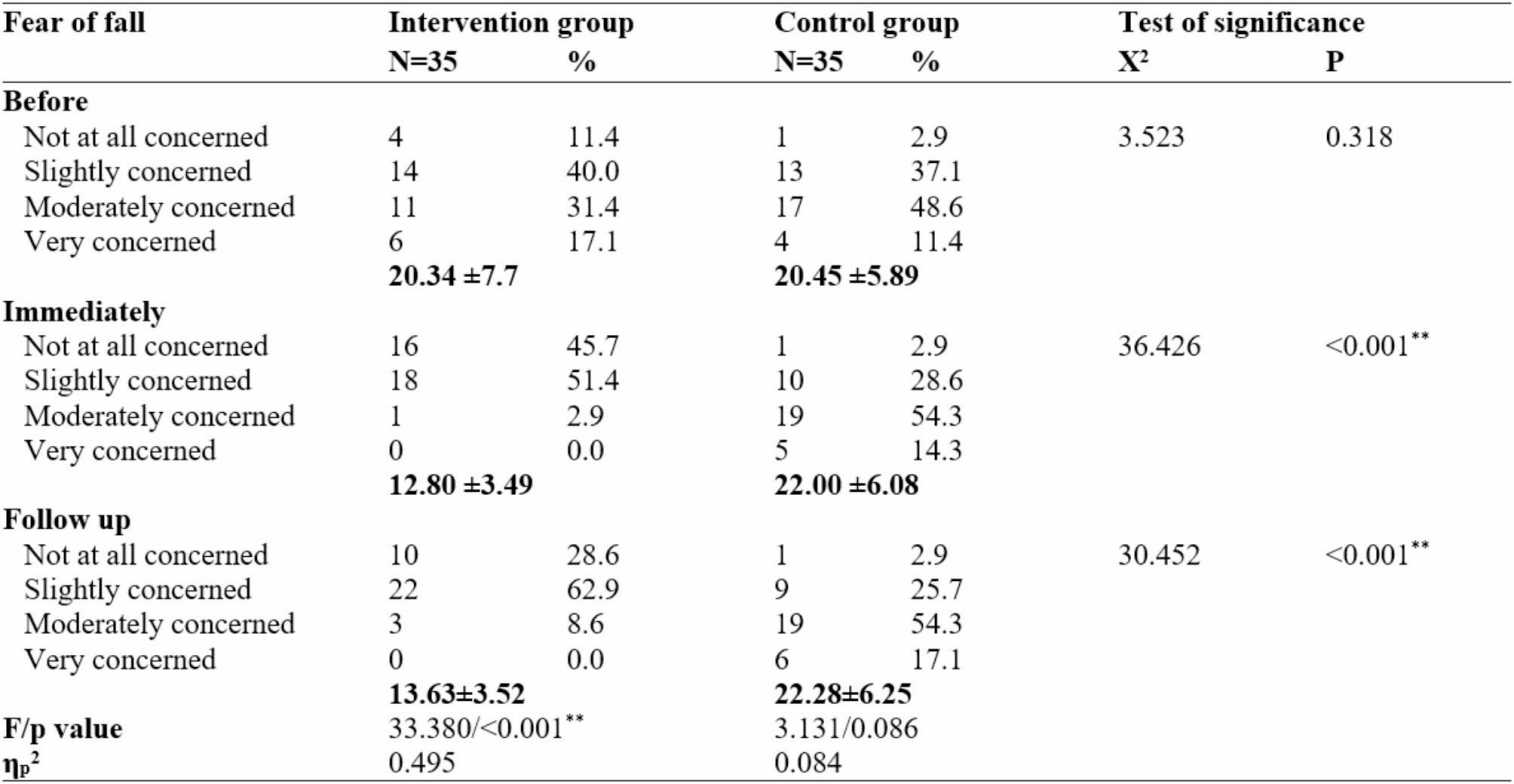
Fear of fall among frail older adults in both groups on three occasions (before, immediately after, and during follow-up).

**Table 5:**
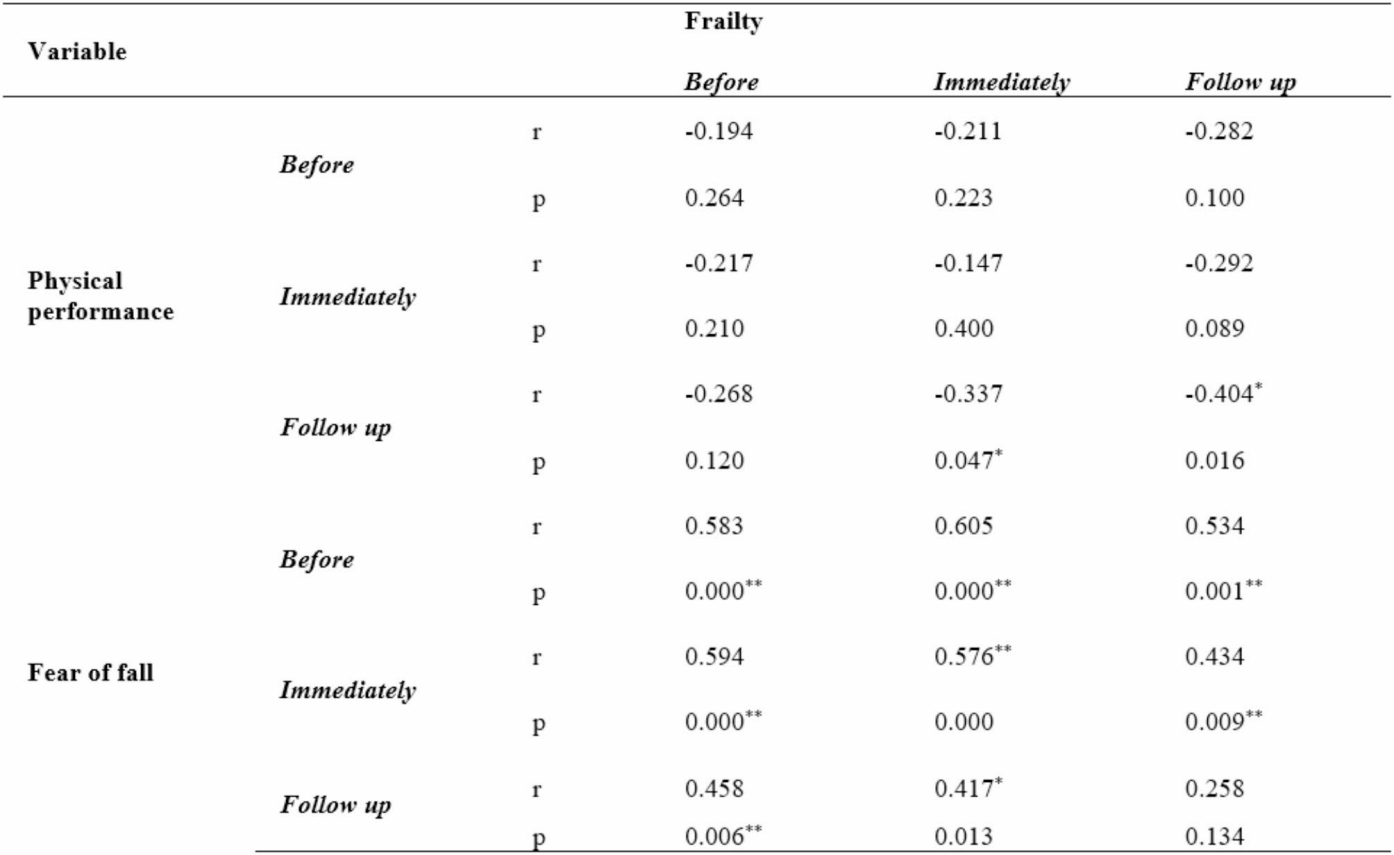
Correlation matrix between the study variables among frail older adults in the intervention group at the three time points (before, immediately after, and during follow-up).

## Discussion

The program has shown that one key tactic for encouraging beneficial aging is physical activity. Virtual reality (VR) therapy offers significant improvements in motor function and overall general health in patients with various conditions. Virtual reality technology is a potential approach to rehabilitation, but there is yet no clear evidence about how it affects older individuals’ frailty^11^.

According to our findings, older persons who live in communities may benefit from a 4-week home-based exergame program in terms of improved physical function and decreased fear of falling. Thus, the development and dissemination of exergames for use at home may broaden the variety of options and activities accessible to senior citizens^25^. Thus, the purpose of this study was to evaluate how a virtual reality-based home-based physical rehabilitation program affected the functional results of older adults who were frail.

Our findings demonstrated that the VR regimen was viable, with no negative effects, maximal adherence (no dropouts), and acceptable usability for weak older persons. These findings align with the existing body of literature^11,26^, It implies that exposure to virtual reality is a good way to encourage physical activity in older persons while maintaining safety and tolerability^27^. To the best of our knowledge, VR has never been used as an exercise facilitation tool in an older adult population, even though multiple research projects have used exercise programs in this age range.

There is some indication of our full sample in the epidemiology statistics, and it was found that males were more prevalent in the intervention and control groups. The greater prevalence of males in both the intervention and control groups in the home-based physical rehabilitation study via virtual reality (VR) for frail older adults can be explained by a few potential factors, such as sex differences in frailty prevalence, as numerous studies have shown that frailty is generally more prevalent among older women than older men. This is likely due to various biological, social, and lifestyle factors, which could be qualified to the specific characteristics of the study setting or recruitment strategies^28^. Like^29^, who has the research on the relationships between knee extension strength and hand grip strength and physical frailty-related variables, found that men were more common in both groups; however, this finding was dissimilar from that of^30^, in which females were more prevalent.

The age of the older adults in the control group ranged from 60 to less than 65 years, while the age of the older adults in the intervention group ranged from 70 years and older. In line with the current results, a randomized controlled trial was conducted by^31–33^ to assess the effectiveness of a multisystem physical exercise intervention for fall prevention and improving quality of life in older individuals at risk of frailty. The results showed that the intervention group’s older adult population was older than the control groups. However, inconsistent with the findings of RCT performed by^34^ The majority of older persons were 66.4 years old in the control group and 66.9 years old in the intervention group when the effects of a home-based, exergaming intervention were evaluated. In line with the results of the present study, a study performed by^35–37^ revealed no differences in demographic or clinical variables at baseline between participants in both groups.

The medical history of frail older adults in both groups was recorded. Most older adults in the intervention and control groups reported ‘yes’ regarding suffering from any disease. Among them, diabetes mellites was among the most prevalent types of chronic diseases. Nonetheless, a quasi-randomized comparative analysis of two exergame therapies was carried out by^30^, who discovered that musculoskeletal issues were among the most common forms of chronic illnesses in older persons after researching the impact of home-based exercise games on cognition and balance.

The association between frailty and drug use has been described repeatedly in the literature, including in a recent review^38,39^. Given that many medications may worsen physical health, it makes sense that there would be a link between such medications and the main components of frailty. Our results concerning the types of medications used indicated that most frail older adults in both groups consumed hypoglycemic agents.

According to our findings, most older adults did not smoke. Likewise, in characterizing the research population’s baseline attributes and based on frailty at baseline^40^, When frailty and medication-related issues were examined among community-dwelling older persons in Europe, it was shown that most frail older adults did not smoke.

Our results showed that there was no statistically significant difference in the medical history of frail older adults between the two groups at baseline. This may be because the intervention and control groups at baseline suggest a few important points, such as an effective randomization process during the study design and homogeneity of the study population in terms of their health status and medical backgrounds, as well as appropriate inclusion/exclusion criteria to ensure that the participants in both groups had similar medical histories and were suitable for the rehabilitation intervention. Similarly, a study by^41^ found no statistically significant variations in the viability and efficacy of a customized home-based motor training program for older persons living in the community.

The reduction in these frailty criteria in the intervention group during the follow-up period can be explained by the potential benefits of the home-based VR physical rehabilitation program, which enhanced bodily performance, as the VR-based rehabilitation program likely helped improve the participants’ muscle strength, endurance, and mobility, leading to reduced slowness in movement and better grip strength. Thus, targeted exercise and increased physical activity may have helped alleviate the feelings of exhaustion and fatigue experienced by frail older adults^42^.

Overheating (exhaustion), weight loss (shrinkage), slowness of movement, lack of physical activity, and inadequate grip strength are the five frailty criteria among frail older persons. These criteria were high prior to the program and decreased throughout the follow-up. Regarding the five frailty criteria, some of them increased in the control group both immediately following the program and throughout follow-up, while other criteria remained unchanged.

Similarly, a study^43^ when older persons in rural southern Korea were given visual-guided exercise programs to measure their physical frailty, the effect of high-speed power training was shown to have a substantial negative impact on their frailty state and scores. This difference was observed between the experimental and control groups.

Similarly, a multicenter randomized controlled trial^12^ assessed the impact of a collaborative exercise program under supervision on the functional performance of institutionalized older persons who were fragile. The results of a 2-way analysis of covariance showed a substantial improvement in between-group analysis following the intervention. However, unlike the outcomes of a multicenter cross-sectional study performed by^44^, who investigated the connection between older persons’ self-reported physical frailty and sensor-based sports participation assessments and found no discernible differences.

Frailty indices of elderlies in both groups on the three occasions (before, immediately after, and during follow-up). Finally, during the follow-up, the participants in the intervention exhibited considerably decreased frailty indices compared to the control group. The partial eta squared values suggested a considerable impact size of the intervention (ηp2 = 0.491).

Consistent with the current results, a study was performed by^45^, who found that there was a significantly greater decrease in the frailty index in the intervention group than in the control group. In addition, the results of a randomized controlled trial performed on prefrail and frail older adults^46^ showed that both intervention groups improved frailty status, with an impact magnitude that was higher than the control groups. Furthermore, there was a significant variation between the two groups in three of the five physical traits associated with the frailty phenotype: lack of activity, impoverished walking speed, and weakness. Exergaming exercise also considerably restored tiredness.

According to our findings, virtually reality-based physical exercise has been promoted as a possible means of enhancing the health of senior citizens in both clinical and nonclinical groups. When used in conjunction with traditional exercises, this type of intervention can help older persons who might not be motivated to perform traditional exercises recover more effectively. Furthermore, getting older adults to engage in physical exercise can be difficult; as a result, creative and unique strategies are needed to grab their interest and encourage adherence^28^.

Our findings showed that prior to the program, there were no statistically significant changes between the intervention and control groups in terms of physical performance dimensions. This may be justified by the increase in physical fitness, which suggested that the home-based VR physical rehabilitation program was effective at improving the overall physical and functional status of the frail older adults in the intervention group^47^. Similarly, a meta-analysis study performed by^9^ revealed that substantial evidence demonstrated that interactive VR training interventions increased lower limb muscle strength, walking speed, and balance. In addition^48^, reported that VR training interventions increased balance.

These findings were also obtained in a study performed by^49^, Researchers investigated the impact of guided remote rehabilitation on the functional performance of older persons living in the community. Their findings raised the possibility that tele-rehabilitation might be a viable substitute for in-person rehabilitation in terms of enhancing functional performance in older adults living in the community.

In addition, meta-analysis was performed by^50^ and demonstrated that engaging in virtual reality exercise significantly improved physical health and total physical aspect scores. However, there were significant advancements in the balance berg scale cut but not in the timed-up-and-go or 8-foot-up-and-go scores. Additionally^51,52^, studied the impact of exercise gaming on older persons in good health and found a statistically significant impact on equilibrium.

On the other hand, in contrast to the above^53^, there was no improvement in balance or gait among older persons who participated in in-home multicomponent exergame training, according to research on the impacts on brain volume and physical function. This might be the case since treatment adherence is one of the most crucial elements in attaining positive outcomes in terms of balance and fall prevention. Given that this population may occasionally become dependent, that they typically struggle with technology, and that treatment sessions are conducted at home, an extended course of treatment may cause participants to lose interest, which will decrease adherence to the program^27^.

Nonetheless, our findings were inconsistent with those of a study performed by^54^, the chair-stand test showed no discernible positive impact, according to that research. These discrepancies in the research results point to the variability of the data that have been published, indicating that exergames may be a good way to encourage older persons to be more physically active. With regard to fear of fall in the intervention and control groups before the program, no statistically significant variation was observed between the groups. The partial eta square in the intervention group (ηp2 = 0.495) indicated that the effect size was large. Similarly, meta-analysis worked by^9^ revealed that substantial evidence demonstrated that interactive VR training interventions reduced fall risk.

According to the outcomes as of right now of the present study, a study performed by^55^ showed that the prevalence of frailty increases with age; Frailty was more common among older adults (70 years of age and above). The following groups were more likely to be frail: women, widows, the illiterate, those who did not work after retirement, those with insufficient monthly income, and those living with someone other than close relatives. On the other hand, a study performed by^56^ indicated that a number of variables, including the degree of education attained in elementary school, had no correlation with the presence of either prefrailty or frailty; as a result, education was included in the final multivariable model as a potential confounder.

In our investigation, there was a strong correlation found between several health variables and prefrail or frail people. Identifying the health indicators that predict frailty and using that information to guide appropriate early intervention points is recommended. Our research results offer proof of the connection between frailty and the medical record of older adults who are feeble.

This may be because a variety of health determinants are linked to frailty and prefrailty status, as certain medical history factors, such as previous hospitalization, smoking, and lack of regular checkups, are predictive of frailty in older adults, and identifying these predictive health factors can inform early intervention strategies to address and potentially prevent frailty in older populations. These findings highlight the importance of considering an individual’s medical history when assessing and managing frailty^57^.

Similarly, a study performed by^55^ revealed that older persons with several diseases, those taking five prescriptions, and those who did not take their medications as prescribed were considerably more likely to be frail. Additionally, there was a strong correlation between frailty and prior hospitalization, smoking, and routine checks.

Further study^56^ conducted a study on ‘frailty and determinants of health among older adults in the United States’ and revealed that many factors were strongly associated with frailty, such as ≥ 2 hospitalizations in the previous year, > 2 comorbidities, and polypharmacy, while factors associated with lower odds of having prefrailty or frailty were being married or living with a partner and not owning a home and age > 80 years, but the effect was small.

The statistically significant relationship between the medical history and physical performance of frail older adults underscores the importance of considering the impact of comorbidities and chronic health conditions when designing and implementing interventions to address frailty and encourage healthy aging and targeted management of chronic diseases and comorbidities may help improve the physical function and performance of frail older adults, potentially delaying or reversing the progression of frailty^57^.

The correlation involving the individual characteristics of frail older adults and their fear of falling was assessed. There was a statistically significant association between the individual characteristics of frail older adults and fear of falling, as sex and marital status; education level; monthly salary (before, immediately and during follow-up); residence (immediately and during follow-up); age (before and immediately); and current work (previously). Similarly, a study by^58^ revealed that sex (being a woman) was a predictor of falls in community-dwelling older adults. Dissimilar to conclusions by^59^, participants who suffered from fear of falling were more prone to be older and less educated than were those who had no fear of falling.

The findings from this study indicate a direct link between a previous medical history and a fear of falling. There was a statistically significant relationship between the medical history of older adults and their fear of falling during the previous year.

A study performed by^59^ revealed that participants who had a fear of falling were more prone to having a number of chronic diseases; Individuals who feared falling experienced higher levels of pain and sadness, were more likely to be admitted to the hospital, engaged in less strenuous activities, and fell more frequently. Additionally, a study by^58^ revealed a relationship between medical history of older adults and fear of falling regarding polypharmacy and a lower SPPB score.

Our results show the correlation matrix between the study variables among frail older adults in the intervention group on the three occasions. A statistically significant weak negative correlation was found between frailty and physical performance, as increasing frailty score was associated with decreasing physical performance score and vice versa. Similarly, in a study performed by [60], The scientists looked at the connection between older individuals’ frailty syndrome and physical performance and discovered a negative correlation between the two.

According to our findings, there was a statistically significant moderate positive association between the fear of falling and frailty (before, just after, and throughout follow-up). Increasing the frailty score was linked to increasing the fear of falling, and vice versa. This might be the case since activity limitation raises the likelihood of falling and vice versa, and both frailty and fear of falling can cause them. Similarly, a study performed by [61] on the factors associated with fear of falling among frail older adults revealed that greater fear of fall is also present in people who are most frail, with several studies describing the relationship between frailty and fear of falling and falls [62]; [63].

Slowness and tiredness were the frailty criteria that in our sample most significantly raised fear of falling ratings. [64] noted a connection as well. Further research should be done on these results as well, as they may indicate that criteria for identifying and preventing frailty need to be adjusted if different frailty criteria have different effects on fear of falling or other outcomes.

This is the first study, to the best of our knowledge, that assessed the impact of a virtual reality-based home-based physical rehabilitation program on the functional outcomes of frail older adults in order to investigate potential mechanisms of intervention effects. Exergaming will be incorporated into the program to support the established advantages of exercise in this susceptible group. We expect to see a drop in the frailty index as well as an increase in standing balance, gait speed, chair standing, and fear of falling.

## Conclusion

The groups’ evaluation findings and baseline characteristics were comparable. Regarding the frailty criteria, standing balance, gait speed, chair stand, and fear of falling, we saw a considerable improvement in the intervention group and no change in the control group. Furthermore, a small but statistically significant negative association was discovered between physical performance and frailty, with a higher physical performance score being linked to a lower frailty score and vice versa, and a moderate positive correlation was shown between frailty and fear of fall as the frailty score increased, which was related with a cumulative fear of fall score and vice versa.

### Recommendations

The study’s conclusions lead to the following suggestions being put forth:

- The potential long-term benefits of the home-based VR rehabilitation program on reducing the risk of hospitalization, institutionalization, and other adverse health outcomes older adults.
- Develop strategies to monitor and promote participant adherence to home-based VR rehabilitation programs, such as regular check-ins, remote monitoring, and troubleshooting support, and assess participants’ level of engagement and enjoyment with VR technology to understand its impact on overall outcomes.

## Data Availability

The datasets used and/or analyzed during the current study are available from the corresponding author upon reasonable request.
